# New SIMS U-Pb age constraints on the largest lake transgression event in the Songliao Basin, NE China

**DOI:** 10.1371/journal.pone.0199507

**Published:** 2018-06-26

**Authors:** Dangpeng Xi, Huaiyu He, Zhiqiang Yu, Qinghua Huang, Jianfang Hu, Yankang Xu, Zhongye Shi, Zuohuan Qin, Xiaoqiao Wan

**Affiliations:** 1 State Key Laboratory of Biogeology and Environmental Geology, China University of Geosciences, Beijing, China; 2 State Key Laboratory of Lithospheric Evolution, Institute of Geology and Geophysics, Chinese Academy of Sciences, Beijing, China; 3 University of Chinese Academy of Sciences, Beijing, China; 4 Exploration and Development Research Institute of Daqing Oil Field Corporation, Daqing, Heilongjiang, China; 5 Guangzhou Institute of Geochemistry, Chinese Academy of Sciences, Guangzhou, Guangdong, China; Tongji University, CHINA

## Abstract

The largest lake transgression event (LTE) associated with lake anoxic events (LAE) and periodic seawater incursion events (SWIE) in the Songliao Basin, northeastern China, occurred during deposition of the Cretaceous Nenjiang Formation. The Yaojia-Nenjiang Formation boundary (YNB) marks the beginning of the LTE, as well as LAE and SWIE. However, there is an absence of direct radioisotopic dating, and therefore the age of the YNB, as well as the beginning of LTE, together with their relationship with other geological events, is strongly debated. Here we present a new SIMS U-Pb zircon age from the lowermost Nenjiang Formation. The bentonite bed located 9.88 m above the YNB of the X1-4 borehole was analyzed. Twenty-five analyses of 25 zircons were conducted, which produced a weighted mean age of 85.5±0.6 Ma (MSWD = 0.87). Based on the average sediment accumulation rate, the age of the YNB is suggested to be 85.7 Ma, indicating that the LTE began in the Early Santonian. The new ages provide a precise chronostratigraphic framework for climatic and geological events. Our new results imply that the beginning of the LTE, LAE and SWIE occurred almost simultaneously with short-term sea level rise, and probably had a close relationship with OAE3.

## Introduction

**Fig 1:**
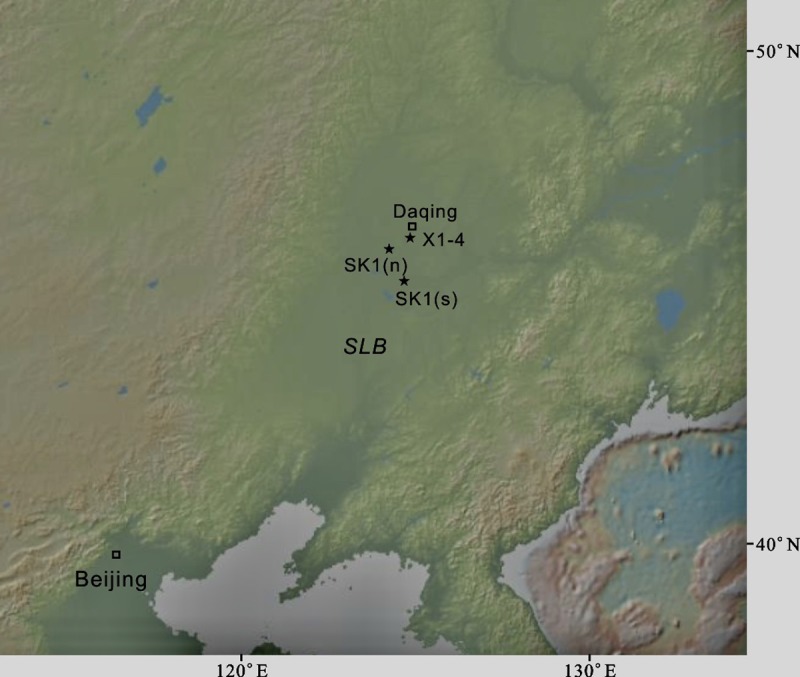
Locations of the study site and the International Continental Scientific Drilling Project (SK-1) in the Songliao Basin (SLB). (map modified from GeoMapApp; http://www.geomapapp.org/) Locations of the X1-4 borehole (124°55'1''N, 46°25'43''E), the north core, SK-1(n) (46°12'44.22''N, 124°15'56.78''E), and the south core, SK-1(s) (45°34'14.42''N, 124°40'15.59''E).

The Cretaceous was one of the warmest periods in Earth history and is characterized by oceanic anoxic events (OAEs), intense volcanic activity, a long period of normal geomagnetic polarity (that is, the Cretaceous Normal Superchron (CNS)), and high sea level [1–6]. Paleoclimatic and paleoenvironmental changes in the Cretaceous ocean have been well studied, but there is relatively little information about the continental record and its associated localized climate changes. Cretaceous continental sedimentary records provide key evidence that can increase our understanding of how terrestrial systems respond to both regional- and global-scale environmental changes [7]. The Songliao Basin (SLB), in northeastern China, is one of the largest Cretaceous continental rift basins in the world [8–10] (Fig 1). The basin was a long-lived and mid-latitude lake basin that was located relatively close to the western Pacific Ocean during the Cretaceous [10–12], and was thus subjected to seawater incursions. The Cretaceous ocean system may have been connected to the SLB through either atmospheric processes or oceanic transgression onto continental margins [13,14]. Well-preserved Cretaceous lacustrine deposits in the basin provide a unique opportunity to study the Cretaceous paleoenvironment and paleoclimate, as well as geological events [11,12,14].

The largest lake transgression event (LTE) in the region occurred during the Late Cretaceous, with dark mudstone, black shale and oil shale preserved in the Lower Nenjiang Formation (K_2_n), resulting in a large-scale lake anoxic event (LAE) [15]. During this event, the Songliao paleolake was also periodically inundated by seawater incursion events (SWIE) [16–23]; as such, the lower K_2_n units may have been affected by changes in both regional and global sea level [24]. Several workers have suggested that this large lake transgression in the SLB was related to an OAE [13,15,25], while others have challenged this view on the basis of the dissimilar timings for each event [26,27]. Recently, Wagreich et al. [28] proposed that the high lake level in the SLB might have been related to a short-term low point on the global sea-level curve. Eustatic changes in sea level during the Cretaceous have been reevaluated using a synthesis of global stratigraphic data [5], which can be used to interpret lake-level changes within the SLB. Detailed stratigraphic data and a precise geochronology allow an improved understanding of the paleoclimate and paleoenvironment that existed within the SLB during the Cretaceous, as well as their relationship with marine and continental rocks of the period.

The International Continental Scientific Drilling Project in SLB (SK-1) spanned the complete sequence of Upper Cretaceous strata [12,29]. Three U-Pb ages were previously obtained from the lower Qingshankou Formation (K_2_qn), and one from the lowermost member 2 of the Nenjiang Formation (K_2_n^2^) of the SK-1 [26,29]. However, no radioisotopic dating has yet been performed on units between the middle K_2_qn and Member 1 of the Nenjiang Formation (K_2_n^1^). The Yaojia Formation/Nenjiang Formation boundary (YNB) marks the beginning of the LTE, as well as LAE and SWIE, but its age is uncertain. The current age of the YNB was estimated to be 84.487 Ma based on magnetostratigraphy [30], U-Pb dating, and the average sediment accumulation rate; or 84.673 Ma based on cyclostratigraphy [27]. Consequently, the age of the YNB is debated, which hinders our understanding of the LTE, LAE, SWIE, as well as their relationship with global-scale climate, lake level and OAE3.

In this study, we report a new age from the lowermost K_2_n^1^ based on investigation of a bentonite layer located 9.88 m above the YNB that was obtained from borehole X1-4 in the Central Depression of the SLB (Fig 1). The significance of the new dating results is as follows. On the one hand, it provides new constraints on the YNB, and improves the chronostratigraphic framework of the lower part of K_2_n^2^ in SLB, which is important for understanding climate change during the early Santonian to early Campanian. On the other hand, as the lower K_2_n^2^ was subjected to SWIE, it was affected by both regional and global events. On the basis of this new chronostratigraphic framework, the local lake level, LAE and global sea level and OAE3 are well correlated.

## Materials and methods

**Fig 2:**
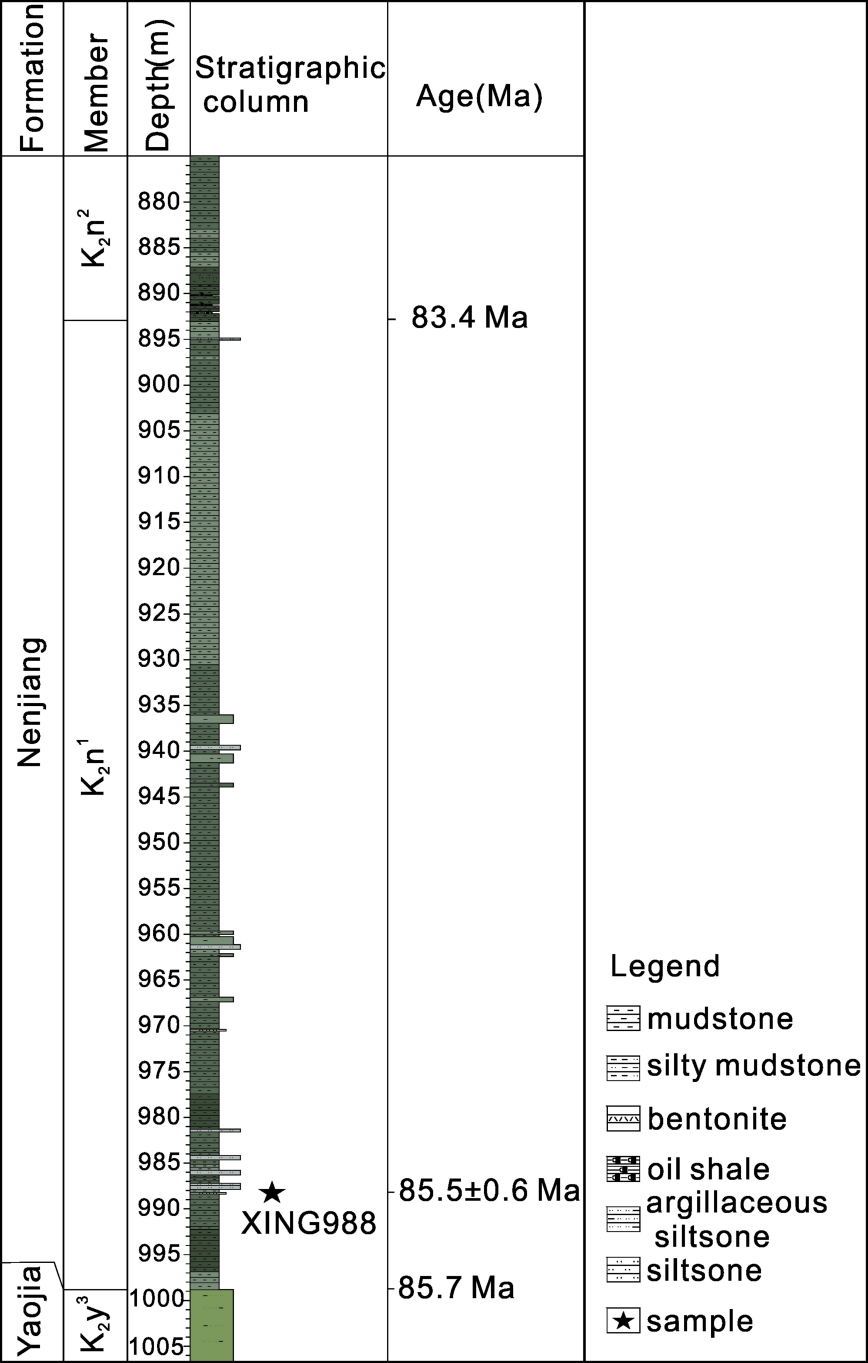
Stratigraphy of borehole X1-4. Fig 2 was created by CorelDRAW X8 (http://www.coreldraw.com/us/pages/free-download). Copyright (c) 2018 [Huaiyu He] and its licensors. All rights reserved.

The Lower Nenjiang Formation (K_2_n^1^ to lower K_2_n^2^) of the X1-4 borehole (124°55'1''N, 46°25'43''E), which is located at Changyuan-Xingshugang area of SLB, has been investigated in this study. The Lower K_2_n is mainly composed of dark mudstone with a bentonite layer at the depth interval of 988.26–988.29 m, where a sample (XING988) was collected (Fig 2). Permits for collecting the sample were not required. The K_2_n^1^/K_2_n^2^ and K_2_y/K_2_n boundaries occur at depths of 892.67 m and 998.17 m, respectively. The sample XING988 used for U-Pb geochronological analysis were processed by conventional magnetic and density techniques in order to concentrate non-magnetic, heavy fractions. Zircon grains obtained from the samples were mounted in epoxy alongside zircon reference standard Plésovice [31] and Qinghu, which were all subsequently polished to half of their thicknesses so that their centers were exposed for isotopic analysis. All zircons were examined using transmitted and reflected light photomicrographs, as well as cathodoluminescence (CL) images in order to reveal their internal structures, and the mount was vacuum-coated with high-purity gold prior to secondary ion mass spectrometry (SIMS) analysis.

Measurements of U, Th, and Pb were conducted using a Cameca IMS-1280 SIMS at the Institute of Geology and Geophysics, Chinese Academy of Sciences in Beijing. U-Th-Pb ratios and absolute elemental abundances were determined relative to the zircon standard 91500 [32], analyses of which were interspersed with those of unknown grains, using operating and data processing procedures similar to those described by Li *et al*. [33]. A long-term uncertainty of 1.5% (1 RSD) for ^206^Pb/^238^U measurements of the standard zircons was propagated to the unknowns [34], despite the measured ^206^Pb/^238^U error in any specific session having been around 1% (1 RSD) or less. Measured compositions were corrected for common Pb using non-radiogenic ^204^Pb. Corrections are sufficiently small to be insensitive to the choice of common Pb composition, and an average of present-day crustal composition [35] was used for the correction, assuming that it largely occurred as surface contamination introduced during sample preparation. Uncertainties of individual analyses in the data tables are reported at the 1σ level, and mean ages for pooled U/Pb and Pb/Pb analyses are given at the 95% confidence interval. SIMS U-Pb zircon data are presented in Table 1. Data reduction was carried out using the Isoplot Excel add-in v. 2.49 [36].

**Table 1:** Secondary ion mass spectrometry zircon U-Pb data.

					Conventional Concordia Columns (Pbc corr.)							
Sample/ spot	[U] (ppm)	[Th] (ppm)	f206%	Th/U meas	^207^Pb/^235^U	±σ (%)	^206^Pb/^238^U	±σ (%)	^207^Pb/^235^U	±σ (%)	^206^Pb/^238^U	±σ (%)
**XING998@1**	**392**	**187**	**{2.42}**	**0.478**	**0.07404**	**9.27**	**0.0132**	**1.60**	**72.5**	**6.5**	**84.6**	**1.3**
**XING998@2**	**571**	**486**	**{0.11}**	**0.852**	**0.08634**	**2.64**	**0.0131**	**1.56**	**84.1**	**2.1**	**84.0**	**1.3**
**XING998@3**	**465**	**296**	**{0.44}**	**0.636**	**0.09058**	**3.55**	**0.0136**	**1.70**	**88.0**	**3.0**	**86.9**	**1.5**
**XING998@4**	**356**	**203**	**{0.15}**	**0.570**	**0.09374**	**3.07**	**0.0133**	**1.54**	**91.0**	**2.7**	**85.2**	**1.3**
**XING998@5**	**537**	**276**	**{0.26}**	**0.514**	**0.08770**	**2.87**	**0.0136**	**1.53**	**85.4**	**2.4**	**86.9**	**1.3**
**XING998@6**	**652**	**405**	**{0.37}**	**0.622**	**0.08862**	**2.19**	**0.0135**	**1.52**	**86.2**	**1.8**	**86.4**	**1.3**
**XING998@7**	**295**	**132**	**{0.41}**	**0.447**	**0.08814**	**2.86**	**0.0134**	**1.55**	**85.8**	**2.4**	**85.9**	**1.3**
**XING998@8**	**376**	**231**	**{3.98}**	**0.615**	**0.09076**	**20.95**	**0.0133**	**1.77**	**88.2**	**17.9**	**85.3**	**1.5**
**XING998@9**	**378**	**151**	**{3.31}**	**0.398**	**0.07236**	**14.23**	**0.0131**	**1.62**	**70.9**	**9.8**	**84.0**	**1.4**
**XING998@10**	**619**	**445**	**{0.26}**	**0.719**	**0.08911**	**2.20**	**0.0135**	**1.50**	**86.7**	**1.8**	**86.5**	**1.3**
**XING998@11**	**388**	**226**	**{2.11}**	**0.582**	**0.07902**	**7.90**	**0.0134**	**1.56**	**77.2**	**5.9**	**85.9**	**1.3**
**XING998@12**	**523**	**341**	**{0.34}**	**0.652**	**0.09202**	**2.44**	**0.0137**	**1.56**	**89.4**	**2.1**	**87.4**	**1.4**
**XING998@13**	**453**	**244**	**{4.10}**	**0.538**	**0.08032**	**10.12**	**0.0130**	**1.59**	**78.4**	**7.7**	**83.1**	**1.3**
**XING998@14**	**664**	**423**	**{0.10}**	**0.637**	**0.08711**	**2.21**	**0.0133**	**1.52**	**84.8**	**1.8**	**84.9**	**1.3**
**XING998@15**	**419**	**216**	**{5.98}**	**0.515**	**0.05087**	**16.96**	**0.0121**	**1.91**	**57.3**	**9.5**	**77.6**	**1.5**
**XING998@16**	**481**	**246**	**{0.24}**	**0.511**	**0.08702**	**2.60**	**0.0132**	**1.55**	**84.7**	**2.1**	**84.8**	**1.3**
**XING998@17**	**413**	**193**	**{1.26}**	**0.466**	**0.07887**	**6.18**	**0.0132**	**1.59**	**77.1**	**4.6**	**84.8**	**1.3**
**XING998@18**	**433**	**303**	**{0.75}**	**0.699**	**0.09145**	**2.55**	**0.0135**	**1.55**	**88.9**	**2.2**	**86.6**	**1.3**
**XING998@19**	**438**	**265**	**{0.41}**	**0.605**	**0.09229**	**2.50**	**0.0136**	**1.53**	**89.6**	**2.1**	**86.8**	**1.3**
**XING998@20**	**543**	**335**	**{0.33}**	**0.617**	**0.08689**	**3.21**	**0.0134**	**1.54**	**84.6**	**2.6**	**85.9**	**1.3**
**XING998@21**	**356**	**285**	**{2.73}**	**0.798**	**0.08454**	**11.33**	**0.0136**	**1.61**	**82.4**	**9.0**	**87.1**	**1.4**
**XING998@22**	**422**	**270**	**{1.54}**	**0.641**	**0.08417**	**5.91**	**0.0129**	**1.56**	**82.1**	**4.7**	**82.9**	**1.3**
**XING998@23**	**945**	**911**	**{3.57}**	**0.964**	**0.07625**	**8.24**	**0.0135**	**1.59**	**74.6**	**5.9**	**86.5**	**1.4**
**XING998@24**	**503**	**299**	**{0.22}**	**0.593**	**0.08819**	**3.68**	**0.0135**	**1.53**	**85.8**	**3.0**	**86.4**	**1.3**
**XING998@25**	**344**	**161**	**{0.95}**	**0.469**	**0.08570**	**5.54**	**0.0133**	**1.66**	**83.5**	**4.5**	**85.4**	**1.4**
**XING998@26**	**366**	**174**	**{0.71}**	**0.476**	**0.08324**	**5.00**	**0.0134**	**1.54**	**81.2**	**3.9**	**85.8**	**1.3**

## Results

**Fig 3:**
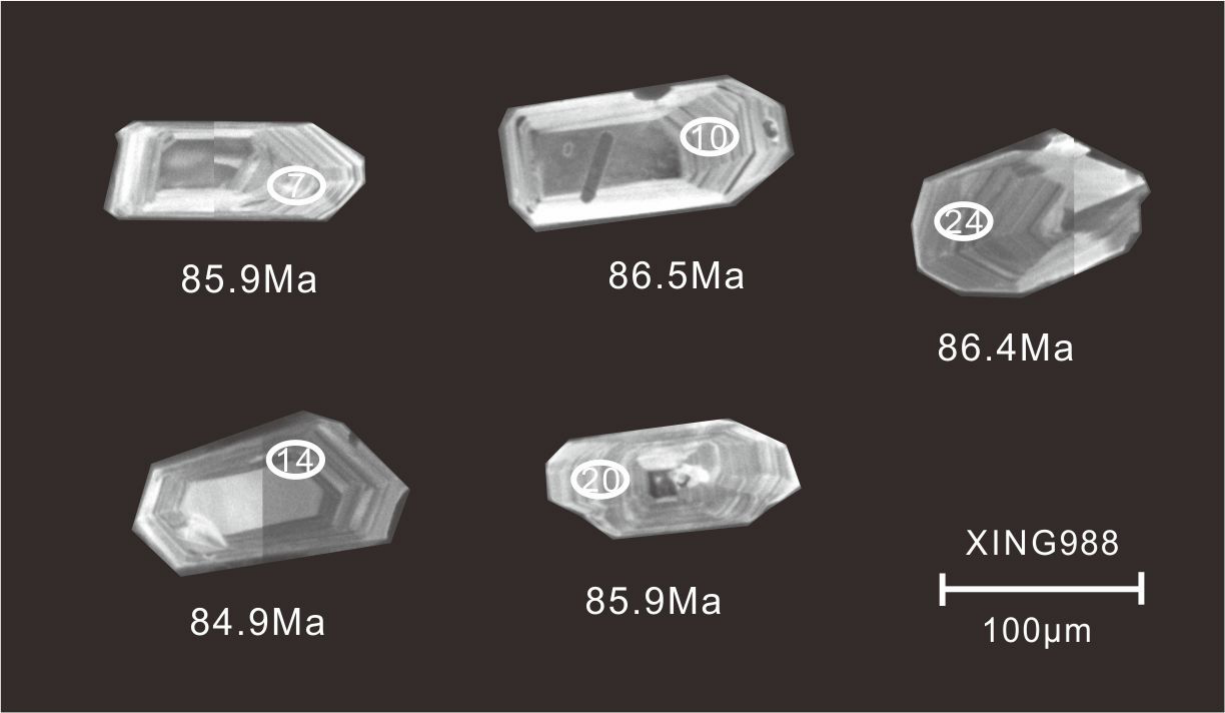
Cathodoluminescence images of representative zircons from sample XING988. Ellipses on the analyzed zircon grains show the positions of U-Pb analytical sites.

Zircons from sample XING988 are mainly euhedral, transparent, colorless, 30–180 μm in length, and have length-to-width ratios between 1:1 and 4:1. Most grains exhibited oscillatory zoning in CL images (Fig 2) and inherited zircon cores were seldom observed. All analyses were conducted on portions of zircon with oscillatory zoning, which is interpreted as reflecting growth within magma. Twenty-five analyses of 25 zircons were obtained in sets of seven scans during a single analytical session. Measured uranium and thorium concentrations were in the ranges 295–945 ppm and 132–911 ppm, respectively, and Th/U ratio varied between 0.4 and 1.0. All analyzed ^206^Pb/^238^U ratios were consistent within the uncertainties associated with the analytical precision. Spot 15 (with distinguishable U-Pb isotopic compositions within analytical errors), gave a younger ^206^Pb/^238^U age of 77.6 ±1.5 Ma. The discordant U-Pb age may be due to the loss of radiogenic Pb. Elimination of this one analytical value left 25 grains which produced a concordant U-Pb age of 85.5±0.6 Ma (Fig 3) and a weighted mean ^206^Pb/^238^U age of 85.5±0.6 Ma (MSWD = 0.87). This age is interpreted as the best estimate of the timing of crystallization of sample XING988.

## Discussion

The Santonian through Early Campanian is an important period during which the global climate began to deteriorate, and numerous significant geological events occurred, such as OAE3, the termination of the CNS [2,28,37]. SIMS zircon analyses of intercalated bentonite layers located at depths of 1019 m (~6 m above K_2_n^1^/K_2_n^2^ boundary) in core SK-1(s) yielded U-Pb ages of 83.7±0.9 Ma [26]. More recently, U-Pb CA-ID-TIMS geochronology performed by Wang et al. [7] produced updated ages of 83.269±0.044 Ma. The U-Pb ages obtained by He et al. [26] using SIMS U-Pb technique, and those obtained by Wang et al. [7] using CA-ID-TIMS U-Pb technique overlap within uncertainties, but the CA-ID-TIMS analyses provide much greater precision [7]. Thus, we use the higher-precision CA-ID-TIMS U-Pb ages in core SK-1(s) [7] to calculate the age of the YNB. The total length of K_2_n^1^ in borehole SK-1(s) is 103.04 m [38], while in borehole X1-4 it is 105.5 m, implying that the sediment accumulation rates at both boreholes were very similar. Given that the bentonite layer from X1-4 is approximately 9.88 m above YNB, and using the ages of bentonite from the K_2_n^2^ of borehole SK-1(s) [7], we suggest that the average sediment accumulation rate for strata of the lower K_2_n^1^ in borehole X1-4 was ~45.5 m/Myr, and the age of the YNB is ca. 85.7 Ma. This indicates that the YNB might be older than the ages obtained from average sediment accumulation rate and astrochronological timescales [27,30]. Combining our new age for the YNB with ages of 85.7 Ma, and 83.269 Ma for strata at the depth of 1019 m within borehole SK-1(s) [7], the average sediment accumulation rate of K_2_n^1^ and the lower part of K_2_n^2^ of SK-1(s) is suggested to be ~44.9 m/Myr, indicating that the age of the K_2_n^1^/K_2_n^2^ boundary is ca. 83.4 Ma. This result provides a new age constraint on the astrochronological timescale and termination of the CNS. The previous astrochronological timescale indicated a duration of 0.791 Myr for K_2_n^1^ [27], roughly corresponding to two 405-kyr eccentricity cycles. However, our new finding suggests a duration of 2.3 Myr for K_2_n^1^, roughly corresponding to five and half 405-kyr eccentricity cycles. In addition, the previous termination of the CNS of SK-1 was estimated based on magnetostratigraphy, isotopic dating of K_2_n^2^, an astrochronological timescale, or on average sediment accumulation rates [7,26,27,30]. The new age provides a more reliable estimate for the average sediment accumulation rate of lower K_2_n of SK-1s, and thus may provide more reliable evidence for the termination of the CNS for future studies. Overall, the new ages provide a more precise chronostratigraphic framework which will contribute to a better understanding of the astrochronological timescale [27], termination of the CNS [26], carbon and oxygen stable isotope composition [39] and paleoclimate [12] of the SLB, as well as of their relationship with global events during the early Santonian to early Campanian.

**Fig 4:**
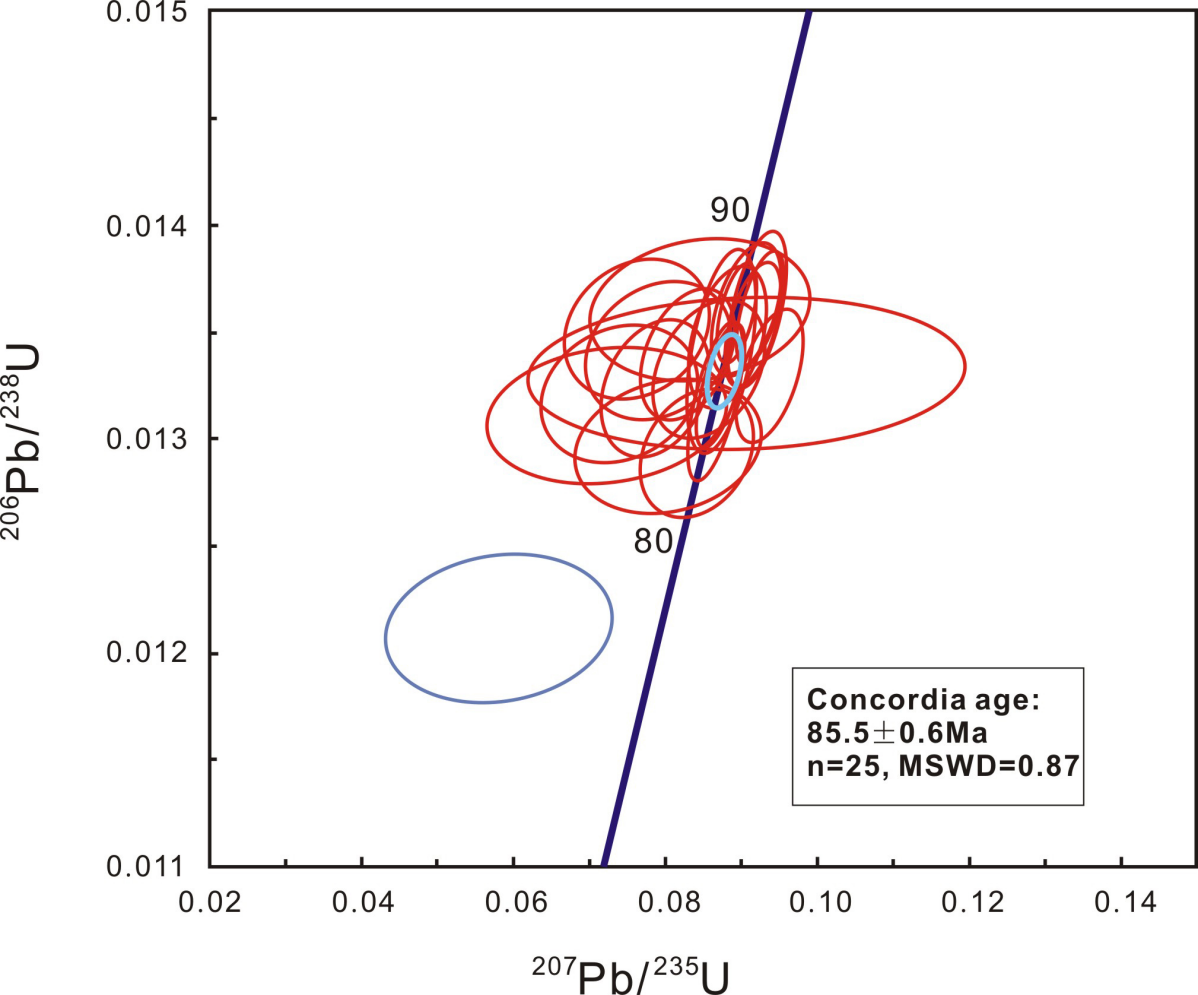
U-Pb concordia diagram showing secondary ion mass spectrometry analytical data for zircons from sample XING988 obtained from a bentonite layer in K_2_n. Data-point error ellipses are 2σ.

Two large LTEs occurred in the SLB during the Late Turonian (lower K_2_qn) and Late Santonian–Early Campanian (lower K_2_n) [15]. The latter event is thought to have been the largest lake transgression to have occurred in the SLB and can be divided into two small stages (Fig 4): Lake transgression event a (LTE2a) during the deposition of lower K_2_n^1^ and b (LTE2b) of lower K_2_n^2^ [13]. During the Lake transgressions, the level of the lake increased and its area expanded [9,15], resulting in lake-wide anoxic events and the formation of productive source rocks for hydrocarbons [8,9,13,14,40–43]^.^ Our new U-Pb age data indicate that the largest lake transgression events began in the Early Santonian at about 85.7 Ma (LTE2a at 85.7 Ma and LTE2b at 83.4 Ma), while two short-term high points on the sea-level curve occurred at 85.5 Ma and 83.2 Ma [5]. This suggests that the beginning of LTE2a, LTE2b, and the short-term rise in sea level occurred almost simultaneously, implying that the LTE documented in the region was closely related to global sea level changes. As the lake level rose and the lake area expanded, as a result of the high global sea level, the seawater likely entered into the Songliao paleolake on a periodic basis [24]. Consequently, a deep lake environment may have formed with oxygen-depleted bottom and brackish waters [14–15,44] resulting in the LAE, which would have provided favorable conditions for the formation of source rocks for hydrocarbons [13,24,43].

Although the relationship between ocean and continental climates during the Cretaceous has long been the subject of intense interest, there is still uncertainty regarding global OAEs and LAE of the SLB. OAE3 occurred during the Coniacian-Santonian [45,46], and the Western Interior Seaway records the classical OAE3, with high organic carbon contents during the Coniacian-Santonian [47]. The Cretaceous SLB has been well correlated and compared with the Western Interior Seaway [42]. Based on a floating orbital time scale, Locklair *et al*. [48] recognized three peaks in organic carbon content during the OAE3 interval, with middle Coniacian, early Santonian and Santonian/Campanian ages. The LAE2 of SLB is closely related to early Santonian OAE3 in terms of age. This may be because both were affected by the relatively high sea level of the Pacific Ocean and the high primary productivity during this period [12–14]. The SWIE of the SLB had the effect of linking the ocean and land together. The relationship between limno-eustasy or aquifer-eustasy has been proposed as a means of studying the Cretaceous climate and ocean circulation [28,49]. From such a perspective, this result implies that there may have been a close relationship between lakes and the ocean during the Cretaceous.

## Conclusions

We have presented a new SIMS U-Pb zircon age from a bentonite bed located 9.88 m above the YNB of the X1-4 borehole. Twenty-five analyses of 25 zircons were obtained which produced a weighted mean age of 85.5±0.6 Ma (MSWD = 0.87). The largest LTE began in the Early Santonian at about 85.7 Ma (LTE2a at 85.7 Ma and LTE2b at 83.4 Ma), while two short-term high points on the sea-level curve occurred at 85.5 Ma and 83.2 Ma. Our new results imply that the beginning of LTE, LAE and SWIE occurred almost simultaneously with a short-term sea level rise, and that they probably had a close relationship with OAE3.
